# Investigating New Applications of a Photoswitchable Fluorescent Norbornadiene as a Multifunctional Probe for Delineation of Amyloid Plaque Polymorphism

**DOI:** 10.1021/acssensors.2c02496

**Published:** 2023-03-22

**Authors:** Ambra Dreos, Junyue Ge, Francisco Najera, Behabitu Ergette Tebikachew, Ezequiel Perez-Inestrosa, Kasper Moth-Poulsen, Kaj Blennow, Henrik Zetterberg, Jörg Hanrieder

**Affiliations:** † Department of Psychiatry and Neurochemistry, Sahlgrenska Academy, University of Gothenburg, 43180 Mölndal, Sweden; ‡ Instituto de Investigación Biomédica de Málaga y Plataforma en Nanomedicina−IBIMA Plataforma Bionand, 29590 Malaga, Spain; § Department of Chemistry and Chemical Engineering, Chalmers University of Technology, 41296 Gothenburg, Sweden; ∥ Departamento de Química Orgánica, Facultad de Ciencias, Universidad de Málaga, 29071 Málaga, Spain; ⊥ Institute of Materials Science of Barcelona, ICMAB-CSIC, Bellaterra, 08193 Barcelona, Spain; # Catalan Institution for Research and Advanced Studies ICREA, Pg. Lluís Companys 23, 08010 Barcelona, Spain; ∇ Clinical Neurochemistry Laboratory, Sahlgrenska University Hospital, 43180 Mölndal, Sweden; ○ Department of Neurodegenerative Disease, Queen Square Institute of Neurology, University College London, London WC1N 3BG, UK; ◆ UK Dementia Research Institute, University College London, London WC1N 3BG, UK; ¶ Hong Kong Center for Neurodegenerative Diseases, Hong Kong 1512-1518, China; & UW Department of Medicine, School of Medicine and Public Health, Madison, Wisconsin 53726, United States; ⬢ Department of Chemical Engineering, Universitat Politecnica de Catalunya, EEBE, Eduard Maristany 10-14, 08019 Barcelona, Spain

**Keywords:** Alzheimer’s disease (AD), amyloid beta (Aβ), amyloid polymorphism, norbornadiene (NBD), photoswitches, multifunctional probes, fluorescence imaging

## Abstract

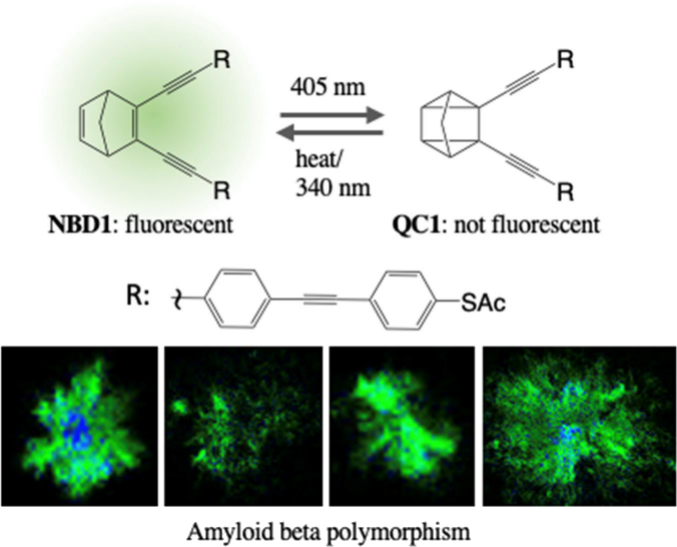

Amyloid beta (Aβ) plaques are a major pathological hallmark of Alzheimer’s disease (AD) and constitute of structurally heterogenic entities (polymorphs) that have been implicated in the phenotypic heterogeneity of AD pathology and pathogenesis. Understanding amyloid aggregation has been a critical limiting factor to gain understanding of AD pathogenesis, ultimately reflected in that the underlying mechanism remains elusive. We identified a fluorescent probe in the form of a turn-off photoswitchable norbornadiene derivative (NBD1) with several microenvironment-sensitive properties that make it relevant for applications within advanced fluorescence imaging, for example, multifunctional imaging. We explored the application of NBD1 for in situ delineation of structurally heterogenic Aβ plaques in transgenic AD mouse models. NBD1 plaque imaging shows characteristic broader emission bands in the periphery and more narrow emission bands in the dense cores of mature cored plaques. Further, we demonstrate in situ photoisomerization of NBD1 to quadricyclane and thermal recovery in single plaques, which is relevant for applications within both functional and super-resolution imaging. This is the first time a norbornadiene photoswitch has been used as a probe for fluorescence imaging of Aβ plaque pathology in situ and that its spectroscopic and switching properties have been studied within the specific environment of senile Aβ plaques. These findings open the way toward new applications of NBD-based photoswitchable fluorescent probes for super-resolution or dual-color imaging and multifunctional microscopy of amyloid plaque heterogeneity. This could allow to visualize Aβ plaques with resolution beyond the diffraction limit, label different plaque types, and gain insights into their physicochemical composition.

Alzheimer’s disease (AD) is the most common neurodegenerative disease^1,2^ though the detailed mechanism of the disease is still not fully understood. The main pathologies observed are the presence of large extracellular plaques consisting of amyloid beta (Aβ) peptides, and intracellular, neurofibrillary tangles (NFTs) composed of hyperphosphorylated tau proteins.^2,3^ Aβ pathology precedes tau and has been suggested to be a critical inducer in AD pathogenesis^3−5^ but the mechanisms that lead to neurodegenerative Aβ and tau pathology in AD are still not clear since some cognitively normal patients also display age-associated extracellular Aβ plaque pathology.^2^ Moreover, Aβ pathology exhibits a large variation in its clinical presentation,^6^ which may relate to varying neurotoxicity of heterogenic, polymorphic plaque phenotypes, such as compact cored deposits and diffuse plaques.^7^ Interestingly, Aβ plaques in cognitively unaffected patients with high amyloid load (cognitively unaffected amyloid-positive, CU-AP) show only diffuse morphology,^8^ suggesting that maturation into cored plaques is a critical feature of AD plaque pathology and AD pathogenesis. It is therefore of key relevance for our understanding of pathogenic amyloid formation in AD to gain insights into the structural and physicochemical characteristics associated with plaque polymorphism. Aβ pathology can be interrogated through fluorescence imaging using small organic molecules.^9−11^ Historically, compounds, such as Congo Red (CR) and thioflavin T (ThT), have been widely utilized; they bind to amyloid cross beta sheets mainly through hydrophobic interactions^12^ and exhibit what is often referred to as aggregation-induced fluorescence (AIF).^12−15^ AIF is induced by the compact environment of the plaques, which can suppress competing radiationless transitions inducing an increase of the fluorescence signal, which further improves visualization of the pathology.^12,16^ The use of small fluorescent molecules as dyes has several advantages; for example, their spectral properties can be affected by the direct binding with amyloid aggregates and interactions with residues in the local microenvironment. This has allowed one to use small fluorescent molecules as multifunctional sensors to harness further information about pathology heterogeneity and development.^17,18^ For example, luminescent-conjugated oligothiophene (LCO) probes have been used to assess different amyloid structures^19^ or to reveal age-dependant polymorphism of Aβ and tau aggregates.^20^ These examples demonstrate how carefully designed small organic molecules can be powerful multifunctional probes to investigate AD pathology. The introduction of photoresponsive fluorescence probes, including ON/OFF fluorescent probes (photoswitches), has had a profound impact on fluorescence imaging techniques making it possible, for example, to acquire images with a resolution higher than the diffraction limit.^21^ Photomodulating the fluorescence, not only between ON and OFF states but also between different colors, can have several advantages; techniques such as dual-color imaging, phase-sensitive lock-in detection, and frequency-domain imaging harness these features to provide improved detection and image quality.^22,23^

Small organic molecules that can be photoswitched between different isomers have been investigated in depth in the recent years.^25^ It is reported how the chemical environment can affect many of the properties such as spectral properties, photoisomerization quantum yields, and kinetics of back-conversion.^26^ For this reason, we speculated that both the spectral and photochemical properties of photoswitchable fluorescent small molecules in amyloid plaques in situ might be affected by steric, polar, or other intermolecular interactions, enabling their use as multifunctional probes to delineate and investigate plaque heterogeneity. Previous efforts using ON/OFF fluorescent probes modified with diarylethene and spiropyran derivatives have been reported for improved detection of Aβ aggregates;^27,28^ the probing moieties in these examples are constituted of already established fluorescent probes, while the photoswitchable moiety is attached with a linker and therefore not necessarily directly interacting with the aggregates. The fluorescent signal could be photomodulated in vitro, in situ, and in vivo. These examples indicate how it is possible and beneficial for several purposes to introduce photoswitchable moieties into small fluorescent molecular probes for AD pathology investigation, and we believe that the concept can be expanded further with different photoswitchable systems.

Norbornadienes (NBDs) are a class of small organic molecules which exhibit photoinduced isomerization to highly strained quadricyclanes (QCs),^29^ and their back-conversion to NBD can be induced thermally or by light irradiation, using a catalyst or even electrochemically.^30−34^ NBD derivatives have been widely explored for a series of applications, including memory storage, solar thermal energy storage, and molecular electronics.^35^ NBD1 is a norbornadiene derivative that bears long conjugated rigid moieties at the 2 and 3 carbon positions (Figure 1a). It was synthesized for being used as a molecular switch in molecular electronics, and its properties in solution (reported in Table 1) are extensively characterized.^24,36^ Moreover, NBD1 is fluorescent, but the photo-induced isomerization breaks the conjugation between the C2 and C3, leading to QC1 which is not fluorescent (Figure 1b).^24^ QC1 to NBD1 isomerization can be triggered both thermally and by means of light irradiation. Therefore, NBD1 exhibits photoswitchable fluorescence which is potentially interesting for several applications within advanced fluorescence imaging techniques such as, for example, super-resolution microscopy or frequency domain imaging (FDI), but it had not been used for imaging applications yet. When observing the molecular structures, we speculated that the long arms with conjugated π systems could possibly interact with the β-sheets of amyloid plaques thanks to hydrophobic interactions. Moreover, the overall molecular structure with its V-shape has been previously reported as a favorable feature in the recognition of Aβ aggregates.^37^ The aim of this work is therefore set to investigate the potential of NBD1 as a photoactive, multifunctional fluorescent probe for amyloid plaques in situ. We were also curious to explore the effects of the interactions with these protein aggregates on the spectral and switching properties of NBD1, in order to harness eventual effects to gain insights into structural and physicochemical heterogeneity within or between plaques.

**Figure 1:**
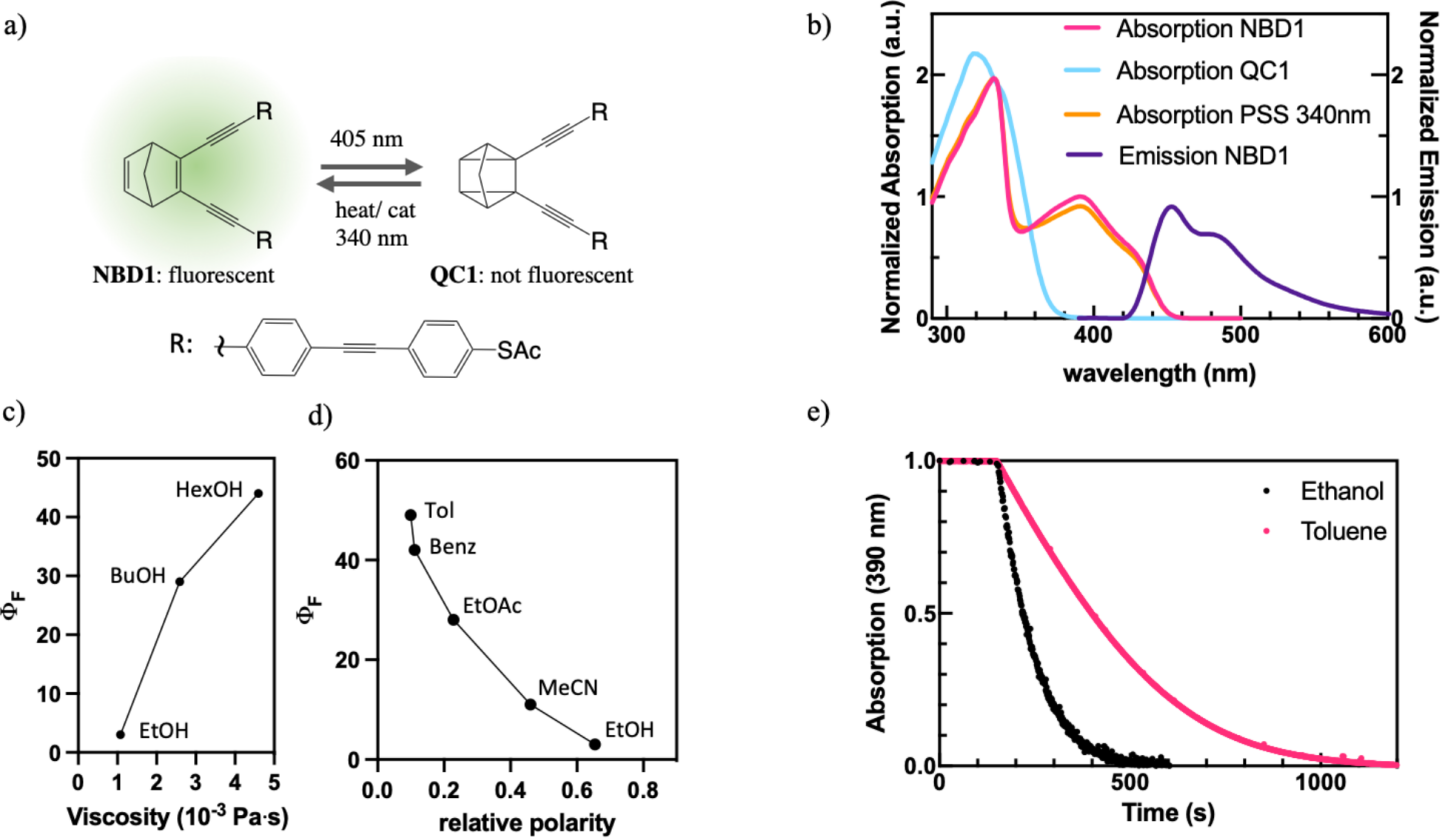
Properties of turn-off fluorescent norbornadiene NBD1: (a) Molecular structure of NBD1 and QC1. Switching of NBD1 to QC1 can be induced by light irradiation, while back conversion to NBD can be induced by heat, light, or a catalyst. (b) Absorption and emission spectra of NBD1 and QC1 in toluene. NBD1 exhibits fluorescence which can be turned off by means of photoinduced isomerization to QC1 with an appropriate light source (here 405 nm). Fluorescence quantum yield is affected by both (c) viscosity and (d) polarity of the solvent. (e) Two solutions of the same concentration of NBD1 in ethanol and toluene subjected by the same irradiation intensity at 405 nm show different photoisomerization efficiencies. NBD1 photoisomerization is more efficient in the more polar solvent, consistent with the opposite trend observed for fluorescence quantum yield, which is a competing process.

**Table 1:** Physicochemical Parameters of NBD1

compound	*t*_1/2 25°C tol_^a^ (min)	ϕ_NBD → QC tol_^b^ (%)	ϕ_f tol_^c^ (%)	ϕ_f benz_^d^ (%)	ϕ_f EtOAc_^e^ (%)	ϕ_f MeCN_^f^ (%)	ϕ_f EtOH_^g^ (%)	ϕ_f BuOH_^h^ (%)	ϕ_f HexOH_^i^ (%)
NBD1	78	15	49	42	28	11	3	29	44

a QC1 to NBD1 isomerization thermal half-life at 25 °C in toluene.^24^

b NBD1 to QC1 photoisomerization quantum yield in toluene.^24^

c Fluorescence quantum yield in toluene.^24^

d Benzene.

e Ethylacetate.

f Acetonitrile.

g Ethanol.

h Butanol.

i Hexanol.

## Results and Discussion

### Effects of Solvent Polarity and Viscosity on NBD1/QC1 Spectroscopic and Physical–Chemical Properties

While NBD1/QC1 has already been fully characterized in toluene solutions, the effects of solvent polarity and viscosity on the spectral and physicochemical properties had not been investigated yet. In order to evaluate these effects, NBD1 absorption and emission spectra were measured in a set of solvents with similar viscosity but a range of relative polarity values (from toluene, with the relative polarity of 0.009 to ethanol, relative polarity 0.654). NBD1 was photoisomerized to QC1 in each of the solvents using a 405 nm diode, and photo back-converted to NBD1 with irradiation at 340 nm (see Figures 1b and S1–S4). The photoisomerization efficiency was also compared between a toluene and ethanol solution with the same concentration and exposed to the same amount of light irradiation, clearly showing that it proceeded more efficiently in the more polar solvent (Figure 1e). This trend was also consistent with the one observed for the fluorescence quantum yield, a competing process, which was highest in the least polar solvents (Figure 1d and Table 1). These experiments indicate that NBD1 photoisomerization and fluorescence efficiencies are affected by the solvent polarity, where a more polar environment favors the photoisomerization, and a less polar environment favors the fluorescence of NBD1. Absorption and emission profiles were also compared in the set of solvents, and both exhibit a slight blue shift in more polar solvents (see SI Figures S5–S7). NBD1 was completely insoluble in commonly used viscous systems such ethylene glycol or glycerol even with the addition of DMSO. Therefore, ethanol, butanol, and hexanol were used to investigate viscosity effects, since they have similar relative polarity but increasing viscosity (from 1.08 to 4.6 × 10^–3^ Pa s). The viscosity of hexanol is much lower than that of glycerol, and cannot simulate the eventual effects of a constrained binding site, but the experiment results were insightful nonetheless. While in this viscosity range, no effects were observed on the absorption and emission profiles, the fluorescence efficiency increased with increasing viscosity (Figure 1c). We can not as for now exclude that there may be other effects when a molecule is constrained in a tight binding pocket.

With being sensitive to solvent polarity and viscosity, we hypothesized that NBD1 has the potential to be employed as a multifunctional probe to label Aβ plaque polymorphism.

### NBD1-Based Fluorescent Imaging of Aβ Plaque Pathology

**Figure 2:**
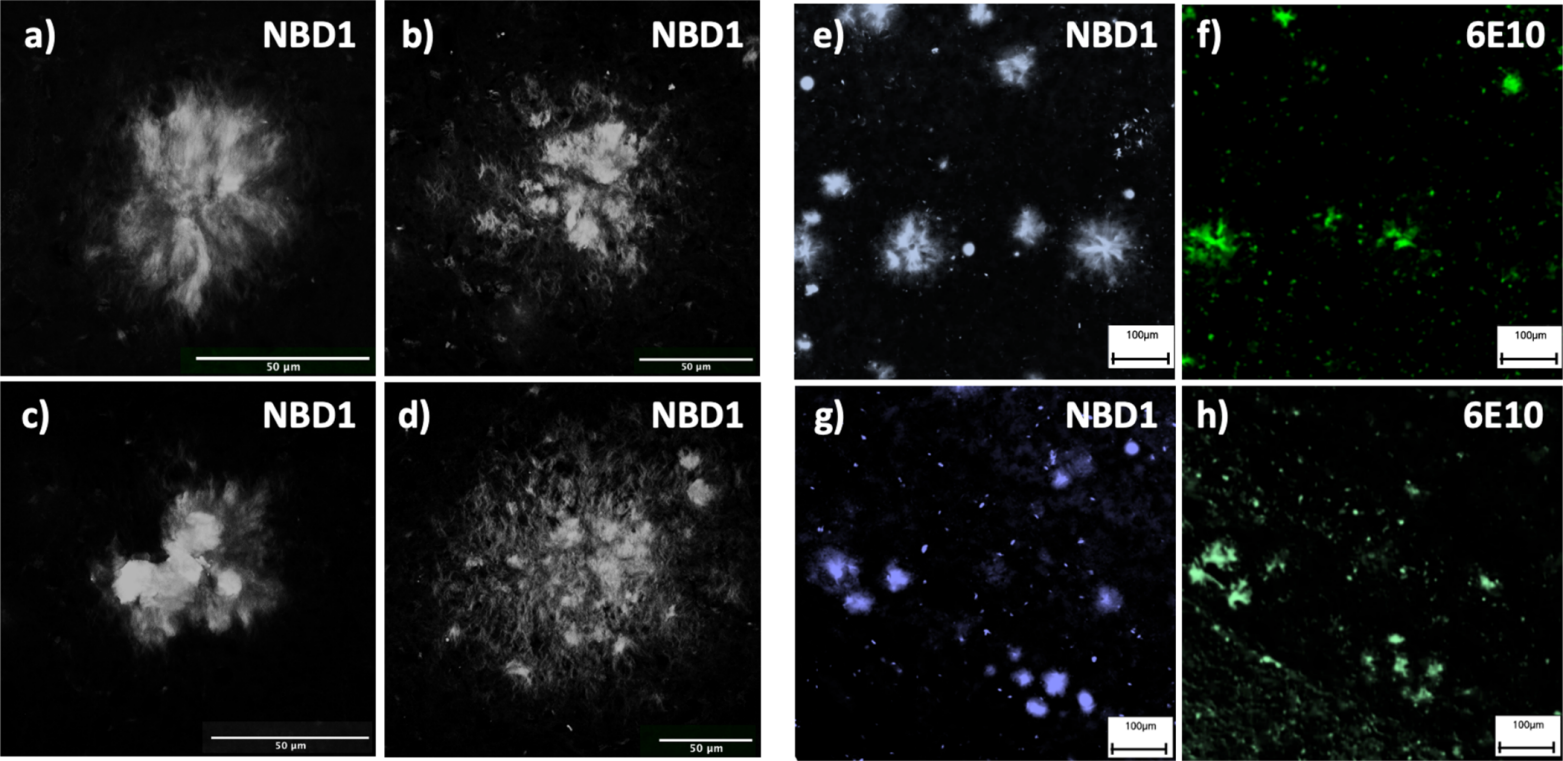
NBD1 as a fluorescent probe for imaging of Aβ pathology in transgenic AD mouse, application, and validation with antibody staining. (a–d) Confocal imaging of cored plaques in the prefrontal cortex of TgAPPSwe 18 months old mice. (e–h) Visualization with widefield microscopy of plaques in the prefrontal cortex (e,f) and hippocampus (g,h) of TgAPPSwe 18 months old mice. The plaques were stained with NBD1 (blue) and 6E10 (green) anti-amyloid beta antibody on consecutive sections and show good co-localization. Scale bars 50 μm (a–d), 100 μm (e–h).

NBD1 is not very soluble in polar solvents, and unfortunately showed poor solubility and stability in different solvent systems commonly used for in vitro Aβ aggregation tests. Therefore, to investigate the ability of NBD1 as a fluorescent stain for Aβ plaques, we decided to directly test it on brain tissue sections of transgenic tgAPPSwe AD mice. Frozen brain tissue sections from 21 or 18 month-old female mice were mounted on microscope slides and after fixation and washing, they were stained with a saturated solution of NBD1 in ethanol for 2 h (detailed method in SI). Already at 18 months, tgAPPSwe mice are characterized by a high plaque load of large cored plaques in both the prefrontal cortex and hippocampus.^38,39^ After NBD1 staining plaques were clearly visible in the blue channel, using both widefield and confocal microscopy (Figure 2a–d).

**Figure 3:**
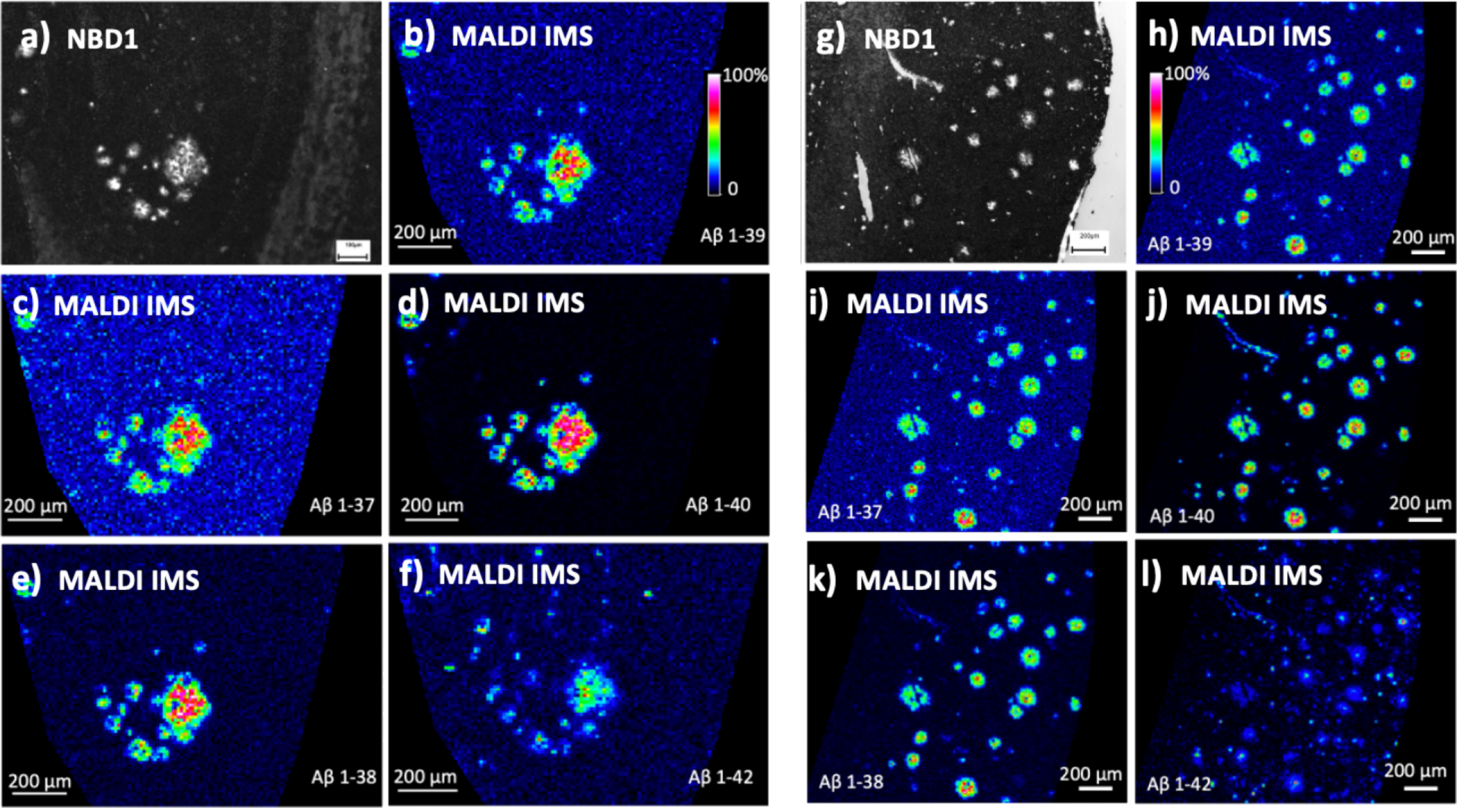
MALDI IMS validation of NBD1 as a fluorescent probe for imaging of Aβ in the transgenic AD mouse brain, validation. NBD1 staining of the hippocampus (a) and prefrontal cortex (g) plaques in TgAPPSwe 18 months old mice. The stainings were visualized with widefield fluorescence microscopy and show co-localization on the same sections with MALDI IMS-detected Aβ peptide isoforms (b–f, h–l). Scale bars 100 (a) or 200 μm (b–l).

To validate the NBD1 staining toward Aβ plaques in situ, the NBD1 imaging data were compared with immunohistochemical (IHC) stainings performed on consecutive sections using the 6E10 Aβ1-16 antibody (Figure 2e–h, detailed methods described in SI). The IHC results showed good co-localization of the detected amyloid plaques with Pearson’s colocalization coefficients of 0.46 in cortical areas and 0.65 in the hippocampus. To further validate and elucidate the Aβ characteristics of NBD1-stained plaques, the tissue sections stained with NBD1 were subsequently analyzed with MALDI imaging mass spectrometry (IMS) (Figure 3). MALDI IMS allows to obtain spatially resolved chemical information about the exact peptide composition of the plaques. Here, plaques in the hippocampus and prefrontal cortex showed a strong colocalization of NBD1 signal with Aβ peptide signals for Aβ 1-37, 1-38, 1-39, 1-40, 1-42. Together these biochemical analyses confirm the selectivity of NBD1 to stain Aβ plaque pathology in situ.

In order to investigate further the binding of NBD1 to Aβ plaques, we performed co-staining of NBD1 and the established hFTAA and qFTAA LCO dyes. It has previously been reported how qFTAA binding increases in the core of mature plaques^40^ Similarily, it has been observed in the present experiments how NBD1 stains qFTAA labeled cores, highlighting NBD1’s preference for the core of mature aggregates (see SI Figures S16 and S17). In addition, NBD1 was also used to stain aggregates in human tissue obtained from sporadic AD (sAD) patients. Here, NBD1 was found to only stain Aβ plaques (see Figure S18). In contrast, LCO stainings of adjacent sections from the same tissue area showed prominent staining of both Aβ plaques and tau tangles that were not visible in the NBD1 staining suggesting that NBD1 shows selectivity toward Aβ plaques. Human AD tissue is unfortunately highly variable with respect to both pathology and condition. Therefore, for the sake of the current study describing NBD1’s amyloid staining properties, we focussed for these experiments on working in transgenic AD mice given their pronounced and exclusive Aβ amyloid pathology.

These results indicate how NBD1 can be used to visualize plaque pathology in tissue using fluorescence microscopy. Moreover, it can be used for correlative multimodal chemical imaging using both light and ion microscopy on the same tissue sections, which can open the way to more in-depth chemical and conformational analyses of plaque composition and development.

### Molecular Docking Studies

**Figure 4:**
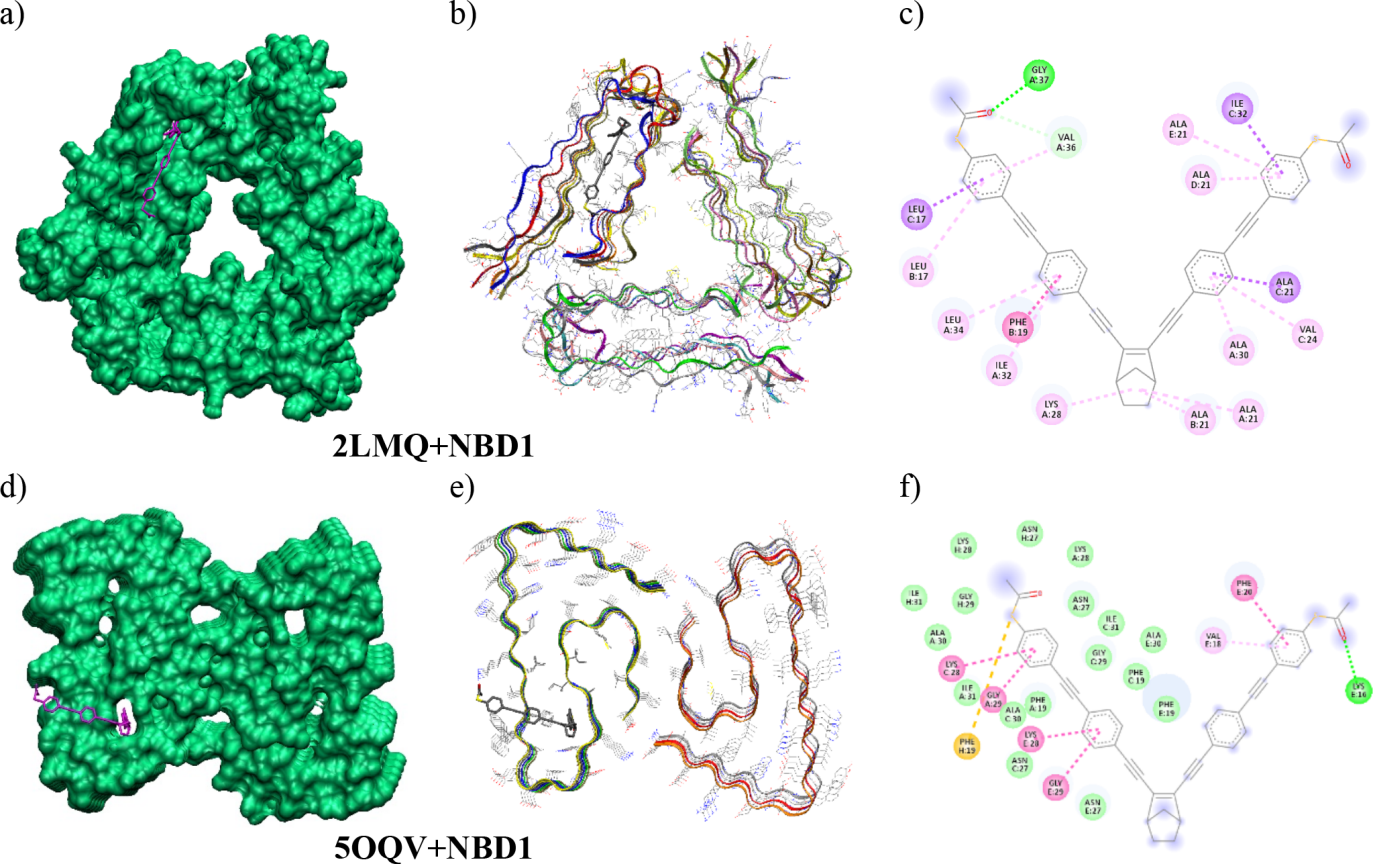
Molecular docking studies: binding of NBD1 and Aβ aggregates. (a–c) Binding modes of NBD1 with the Aβ1-40 (PDB ID: 2LMQ) and (d–f) with the Aβ1-42 (PDB ID: 5OQV) amyloid plaques. (a) 3D image of NBD1-2LMQ interaction in the internal tunnel (surface of the receptor in green and ligand in magenta). (b) 3D image of NBD1-2LMQ interaction (receptor represented as ribbons and ligand with carbons in gray, oxygen in red, and sulfur in yellow. Hydrogens are omitted). (c) 2D interaction diagram of the interactions of NBD1 with 2LMQ. (d) 3D image of NBD1-5OQV interaction in the internal tunnel (surface of the receptor in green and ligand in magenta). (e) 3D image of NBD1-5OQV interaction (receptor represented as ribbons and ligand with carbons in gray, oxygen in red, and sulfur in yellow. Hydrogens are omitted). (f) 2D interaction diagram of the interactions of NBD1 with 5OQV.

Molecular docking simulations were done to evaluate the interactions of NBD1 with Aβ1-40 and Aβ1-42 amyloid plaques. The former was performed using the solid-state NMR structure (PDB ID: 2LMQ) and the later using the cryo-electron microscopy structure (PDB ID: 5OQV). The binding affinity obtained was slightly higher, with a −11.9 kcal/mol docking score, for the Aβ1-40 (2LMQ) amyloid compared to the −10.0 kcal/mol binding affinity for the Aβ1-42 (5OQV) structure. For both amyloid structures, NBD1 places one of its arms inside a tunnel aligned along the fibril axes (Figure 4). The internal cavity of these tunnels has a hydrophobic character and is mainly composed of nonpolar amino acids: Gly, Ala, Val, Leu, Ile, Phe, (see Table S1 for a more detailed characterization of the chains and positions to which these residues belong and Figure S19 for the close contacts between NBD1 and the receptors in SI). This is a well-described behavior for the interactions of some chromophores, based on a curcumin scaffold, with amyloid fibrils.^11,27^ The other arm of NBD1 is placed on a groove in the hydrophilic surface of the fibril axis where are placed polar amino acid residues as Lys and Asn (see Table S1 and Figure S19). The results of molecular docking reflect the interactions between the ligand and the amyloid receptors. One of the NBD1 arms is inside an apolar tunnel while the other arm has a polar environment. This may induce the significant enhancement of the photophysical properties of NBD1 detected in the hyperspectral imaging.

### NBD1-Based Hyperspectral Imaging To Delineate Amyloid Heterogeneity

Following the demonstration and validation of NBD1-based amyloid plaque imaging, we went on to characterize the spectral properties of NBD1 in tissue using hyperspectral confocal microscopy. These experiments were performed on a confocal microscope equipped with a multichannel spectral detector that allowed the collection of image signals at regular intervals of 9 nm between 400 and 600 nm, providing spectral information on each pixel of the image. After the collection of hyperspectral confocal images of large portions of the NBD1-stained AD mouse brain sections, which were including several plaques, the spectra were analyzed by looking at averaged areas of 5 pixel sampling core, periphery, and background areas. The emission band on the plaques was in the range of 420–540 nm, well blue shifted comparing to the emission reported in the toluene solution.^24^

**Figure 5:**
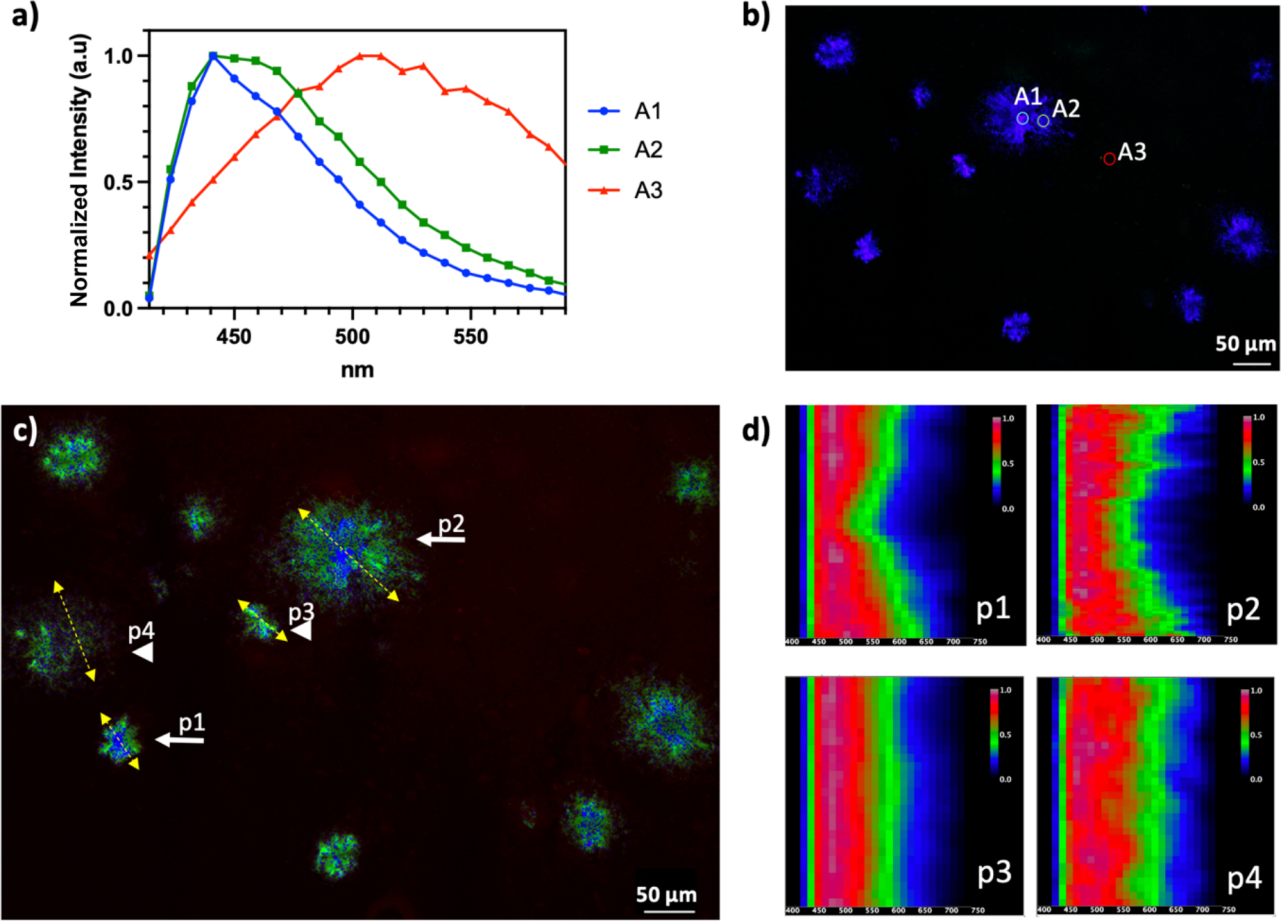
NBD1 probes for Aβ in the transgenic AD mouse brain: hyperspectral confocal imaging and spectral unmixing. (a) In situ-averaged spectra of the plaque core (blue), periphery (green), and background (red), 5 pixel areas sampled as shown in (b). (c) Unmixing of the spectra as shown in (a). (d) Line scan spectral analyses of plaques 1–4 show distinct patterns for cored plaques (p1, p2). Scale bars 50 μm.

The emission band also changed width in different areas of the plaques; in the cases where highly bright and dense cores were observed the emission band was narrower, while in the periphery, it was broader (Figure 5a–c). The observed plaque aggregates do not constitute a homogeneous system; therefore, what we see is possibly the average of different possible binding modes and emission profiles. The trends from the averaged areas samples were used as an indication for the software to identify three spectra through automatic component extraction (ACE). The three extracted spectra are similar to the ones identified by selected averaged areas, and after performing linear unmixing, it was possible to see their emission fingerprint similarly localized in core, periphery, and background areas (see Figure S8). Line scan analyses were performed across several different plaques. These analyses show the emission intensity (where red is the highest and blue is the lowest) over the measured spectrum (400–600 nm) along the drawn lines. Line scans showed a distinct pattern for large, mature, and highly dense cored plaques with a distinct narrower emission band in the dense core, while broader emission bands were confirmed through the whole diffuse plaques and young compact plaques (Figure 5d). Based on the investigations performed in solution, this observed blue shift of emission could indicate interactions with polar residues. From the molecular docking studies, we see how the long rigid conjugated “arm” of NBD1 is inserted inside a pocket (therefore sterically constrained), and the molecule interacts with both polar and nonpolar residues, inside and outside the pocket. It is therefore reasonable to also keep into account the fact that NBD1 is highly constrained in the binding pocket, but unfortunately highly viscous NBD1 solutions could not be characterized due to solubility limitations. Based on the available information, we believe the observed spectral effects are a combination of both steric and polar effects, and what is interesting is to note how the spectral effects correlate with dense cores and peripheries of the imaged plaques. The presented results demonstrate how NBD1 can be used as a single, multifunctional probe to delineate amyloid polymorphism in brain tissue.

### NBD1 In Situ Photoisomerization

**Figure 6:**
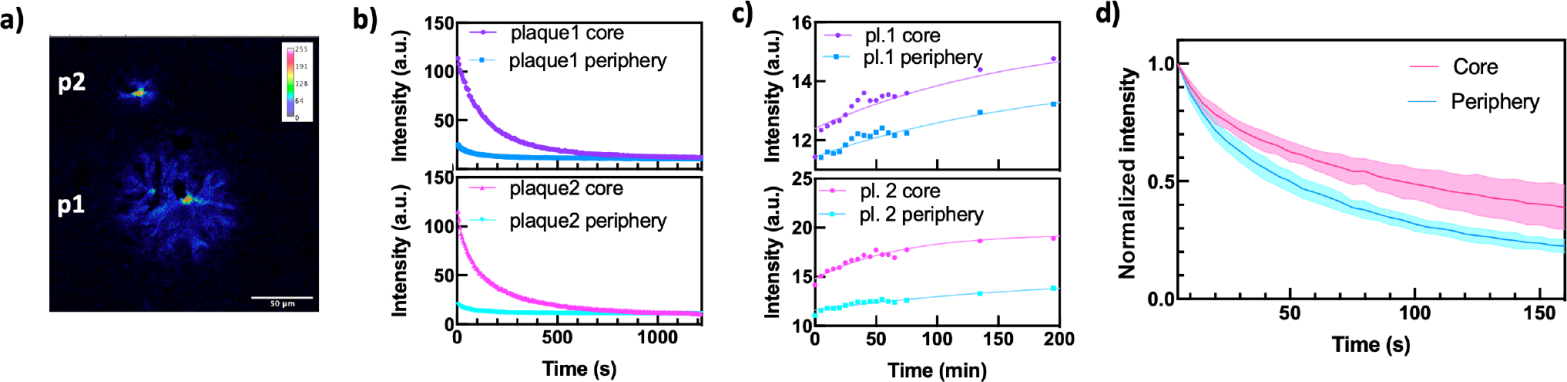
NBD1 as a fluorescent probe for Aβ in the transgenic AD mouse: in situ photoisomerization and back-conversion: (a) Confocal microscopy image of two mature cored plaques stained with NBD1. Intensity of the signal is mapped, and the blue area is considered as periphery, while the red area is the core of the plaques. Scale bar 50 μm. (b) Photoisomerization experiment, fading of fluorescence after irradiation at 405 nm of core (red) and periphery (blue) areas. (c) Thermal recovery over 200 min of the fluorescence signal of plaques 1 and 2, indicating conversion of QC1 to the fluorescent NBD1. (d) Observed fading rates in the core are slower than the ones in the periphery of plaques (reported mean value over 5 plaques, error is shown as light pink or blue area, and it was calculated to be 95% CI).

Finally, we aimed to understand the photoswitching properties of NBD1 in situ. This is relevant for several potential applications of NBDs, such as super-resolution or other advanced imaging techniques, and it is also our interest to investigate the eventual effects of the binding to the photoswitching properties. Here, NBD1-stained AD mouse brain sections were irradiated during the confocal microscope experiment that was set to provide relatively intense light irradiation at 405 nm, followed by image acquisition, every 5 s. The fluorescence intensity was followed over time, and the plaque signal decreased for about 20 min, after which it reached a plateau (Figure 6a,b and Figures S9-S13). Subsequently, the intense irradiation was stopped and images were acquired at regular intervals (of 5 and then 60 min) over 1000 min (Figure 6c and Figure S14, S15). The plaque signal increased on average 27% after 240 min, and 35% after 1000 min, indicating back-conversion of QC1 to the fluorescent NBD1 in situ. In a subsequent experiment, the plaque signals were bleached to about 50% of the initial value in order to assess degradation effects. Similarly, the plaque signal increased by an average of 24% after 240 min.

The experimentally observed decrease in fluorescence signal over the time course of the experiment is generally addressed as “fading”, and it can be in this case affected for example by binding density, photoisomerization of NBD1 to nonemitting QC1, radiation-less relaxation, and degradation. Unfortunately, in this specific experiment, the binding density of NBD1 in the imaged areas could not be controlled or addressed. Nonetheless, it is interesting to see how the fading rates change in the different plaque environments. Signal fading rates over different areas were analyzed, and core and periphery areas averaged intensities were extracted (core and periphery areas are colored in red and blue, respectively, Figure 6a) based on their initial intensity profile. Interestingly, when the signal was normalized (using min-max normalization method), a clear correlation was observed where averaged signals of plaque periphery are fading at a faster rate, while plaque cores are fading at a slower rate (mean values over 5 plaques, and errors calculated as 95% CI are shown in Figure 6d). We can hypothesize how different fading rates in the core and periphery areas could be affected by different NBD1 binding densities or different efficiencies of the photoisomerization and fluorescence competing processes in these areas. Considering that the core of the plaques is known to be very compact, this or other interactions with eventual polar residues in the local microenvironment might lead to, for example, more constrained conformations and therefore hindered photoisomerization, or reduced degradation and radiation-less relaxation. The fact that the fluorescence signal is more intense in the plaque cores is coherent with the hypothesis the competing photoisomerization process might be less efficient. The photoisomerization of NBDs to QCs is known to be accompanied by minor structural changes, and the substituents on C2, C3 are reported to slightly change in orientation;^36^ therefore, a constrained binding site within the compact β-sheets of the plaque core might hinder the photoisomerization process. Further investigations in vitro and in situ will be necessary to gain insights into detailed effects of binding to the photoisomerization process of NBDs and are currently the object of ongoing work. Once the dye is modified to make it water soluble, in vitro measurements while binding Aβ fibrils will provide interesting information. This is, to our knowledge, the first reported photoisomerization and thermal back-conversion of a fluorescent NBD derivative that has been measured inside amyloid plaque pathology in brain tissue. Based on these first observations, we speculate that appropriately optimized NBD systems have the potential to visualize plaques beyond the diffraction limit, label plaque heterogeneity, and advance in-depth knowledge of plaque formation and composition by giving insights into their physicochemical composition.

## Conclusions

In the presented work, a photoswitchable fluorescent norbornadiene derivative, NBD1, was identified as a potential imaging probe for targeting Aβ plaque pathology in situ. NBD1 staining of AD mice brains allowed one to image plaque pathology, and colocalization with IHC and MALDI MSI validates the ability of NBD1 to target Aβ plaques in the AD mouse model. Molecular docking simulations show how one of the rigid conjugated moieties of NBD1 binds the Aβ plaque by insertion into a hydrophobic pocket and highlight interactions with both polar and nonpolar residues; this chemical motif and binding mode are completely novel and offer a new versatile approach to target Aβ plaques. Selectivity in situ experiments suggest the preference of NBD1 toward mature cored Aβ aggregates. Hyperspectral confocal imaging revealed how the spectral properties of NBD1 change in correlation with plaque pathology, exhibiting narrower emission spectra in dense cores of mature plaques, and broader spectra in peripheries and diffused plaques. Photoisomerization and back-conversion experiments in situ were performed for the first time with an NBD derivative, and the observed fading rates were different slower in the cores than in the peripheries of the plaques. Our future focus will be to optimize NBD1 for bioimaging applications, by increasing water solubility, red-shifting absorption and emission, and increasing back-conversion time. These could be addressed in tailored systems, for example, with added solubilizing moieties and extended push–pull π systems which would, based on the available knowledge, both red-shift the spectra and increase back-conversion rates. Overall, the presented work shows a new intriguing opportunity for using tailored photoswitches within advanced imaging of amyloid β pathology in situ, and we hope to develop the concept further in future studies as well toward in vivo applications.

## Supporting Information Available

The SI contains: Figures S1-S16, Tablse S1 and experimental details on spectroscopy measurements, sample preparations, staining procedures, MALDI MS imaging, fluorescent imaging, photoisomerization in situ, human tissue staining, NBD1 and LCO co-stainings, molecular docking, and absorption spectra, emission profiles, automatic component extraction, intensity profiles, and widefield imaging(PDF)

## Notes

The authors declare the following competing financial interest(s): H.Z. has served at scientific advisory boards and/or as a consultant for Abbvie, Acumen, Alector, Alzinova, ALZPath, Annexon, Apellis, Artery Therapeutics, AZTherapies, CogRx, Denali, Eisai, Nervgen, Novo Nordisk, Passage Bio, Pinteon Therapeutics, Prothena, Red Abbey Labs, reMYND, Roche, Samumed, Siemens Healthineers, Triplet Therapeutics, and Wave, has given lectures in symposia sponsored by Cellectricon, Fujirebio, Alzecure, Biogen, and Roche, and is a co-founder of Brain Biomarker Solutions in Gothenburg AB (BBS), which is a part of the GU Ventures Incubator Program (outside submitted work). K.B. has served as a consultant, at advisory boards, or at data monitoring committees for Abcam, Axon, BioArctic, Biogen, JOMDD/Shimadzu. Julius Clinical, Lilly, MagQu, Novartis, Ono Pharma, Pharmatrophix, Prothena, Roche Diagnostics, and Siemens Healthineers, and is a co-founder of Brain Biomarker Solutions in Gothenburg AB (BBS), which is a part of the GU Ventures Incubator Program, outside the work presented in this paper.

## ABBREVIATIONS

AD: Alzheimers disease
Aβ: beta-amyloid
MALDI: matrix assisted laser desorption/ionization
MS: mass spectrometry
NBD: norbornadiene
QC: quadricyclane
tol: toluene
benz: benzene
EtOAc: ethyl acetate
MeCN: acetonitrile
