# Supplementary material for: Investigating New Applications of a Photoswitchable Fluorescent Norbornadiene as a Multifunctional Probe for Delineation of Amyloid Plaque Polymorphism
Source: ACS Sens. 2023 Mar 22;8(4):1500–9. doi: 10.1021/acssensors.2c02496 (PMC10152485; doi:10.1021/acssensors.2c02496)
Supplement: Supplementary file 1 — se2c02496_si_001.pdf [file se2c02496_si_001.pdf]

Supporting Information

Investigating New Applications of a Photoswitchable Fluorescent Norbornadiene as a Multifunctional Probe for Delineation of Amyloid Plaque Polymorphism

Ambra Dreos[1,2*], Junyue Ge[1], Francisco Najera[2,4], Behabitu Ergette Tebikachew[3], Ezequiel Perez-Inestrosa[2,4], Kasper Moth-Poulsen[3,5,6], Kaj Blennow[1,7], Henrik Zetterberg [1,7,8,9,10], Jörg Hanrieder [1,8*]

1 Department of Psychiatry and Neurochemistry, Sahlgrenska Academy, University of Gothenburg, 43180 Mölndal, Sweden; 2 Instituto de Investigación Biomédica de Málaga y Plataforma en Nanomedicina–IBIMA Plataforma Bionand, 29590, Malaga, Spain; 3 Department of Chemistry and Chemical Engineering, Chalmers University of Technology, 41296 Gothenburg, Sweden; 4 Departamento de Química Orgánica. Facultad de Ciencias. Universidad de Málaga. 29071 Málaga, Spain; 5 Institute of Materials Science of Barcelona, ICMAB-CSIC, 08193, Bellaterra. Barcelona, Spain; 6 Catalan Institution for Research and Advanced Studies ICREA, Pg. Lluís Companys 23, 08010 Barcelona, Spain; 7 Clinical Neurochemistry Laboratory, Sahlgrenska University Hospital, 43180 Mölndal, Sweden; 8 Department of Neurodegenerative Disease, Queen Square Institute of Neurology, University College London, London, WC1N 3BG, UK; 9 UK Dementia Research Institute, University College London, London WC1N 3BG, UK; 10 Hong Kong Center for Neurodegenerativa Diseases, Hong Kong, 1512-1518 China; 11 UW Department of Medicine, School of Medicine and Public Health, Madison, WI 53726, USA.

** to whom correspondence should be addressed:*
Dr. Ambra Dreos ambra.dreos@gu.se and Dr. Jörg Hanrieder, jh@gu.se

Index:

1. **Chemical and reagents**
2. **Spectroscopy**
3. **Absorption and emission spectra in different solvents**
4. **Tissue samples preparations**
5. **Fluorescent Staining procedures**
6. **MALDI MS Imaging**
7. **Fluorescent imaging**
8. **Confocal hyperspectral imaging**
9. **Photoisomerization and back conversion *in situ***
10. **NBD1 and LCO co-staining in TgSwe mice tissue**
11. **NBD1 staining of sporadic AD human tissue**
12. **Molecular Docking**

1. Chemicals and Reagents:

All chemicals for matrix and solvent preparation were pro-analysis grade and purchased from Sigma-Aldrich/Merck, unless otherwise specified. TissueTek optimal cutting temperature (OCT) compound was purchased from Sakura Finetek. Deionized water was obtained by a Milli-Q purification system (Millipore Corporation, Merck).
NGS, BSA and Triton were obtained from Sigma, 6E10 (Invitrogen, Thermo Scientific).
Alexa fluor 488 was purchased from Thermo Fisher Scientific.

2. Spectroscopy:

Absorption UV-Vis spectra were measured on a Cary 100 spectrophotometer. Thorlab collimate LED 405 nm and 340 nm were used for irradiations.
Fluorescence measurements were carried out in an Edinburgh Instruments FLS920 fluorometer, equipped with a Xe900 400W Xe lamp as excitation source and R928P photomultiplier detector using quartz cuvettes with 1 cm pathlength.
Absolute quantum yields were measured using an Edinburgh Instruments 1-M-1 integrating sphere using pure solvent as blank.

3. Absorption and emission spectra in different solvents:

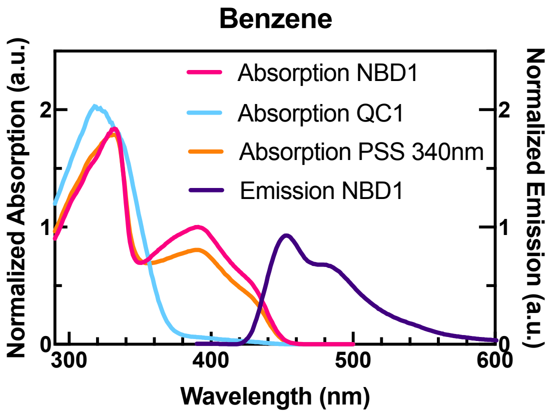

Figure S1: Absorption spectra of NBD1, QC1 (obtained by irradiation at 405 nm), and the photostationary state (irradiation at 340 nm). Emission spectrum of NBD1. All spectra recorded in benzene.

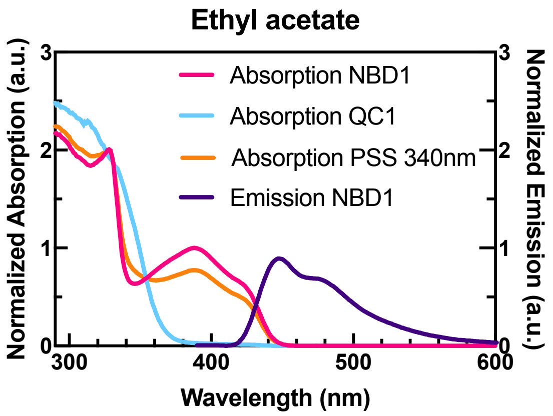

Figure S2: Absorption spectra of NBD1, QC1 (obtained by irradiation at 405 nm), and the photostationary state (irradiation at 340 nm). Emission spectrum of NBD1. All spectra recorded in ethyl acetate.

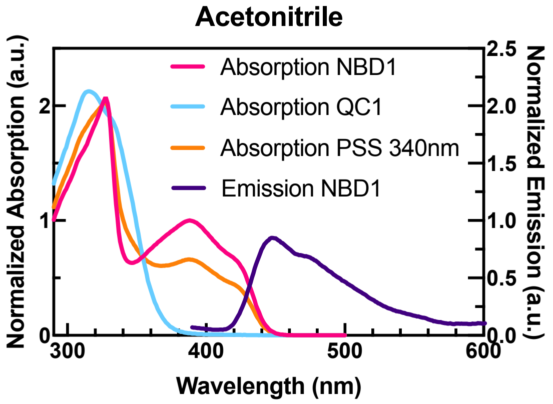

Figure S3: Absorption spectra of NBD1, QC1 (obtained by irradiation at 405 nm), and the photostationary state (irradiation at 340 nm). Emission spectrum of NBD1. All spectra recorded in acetonitrile.

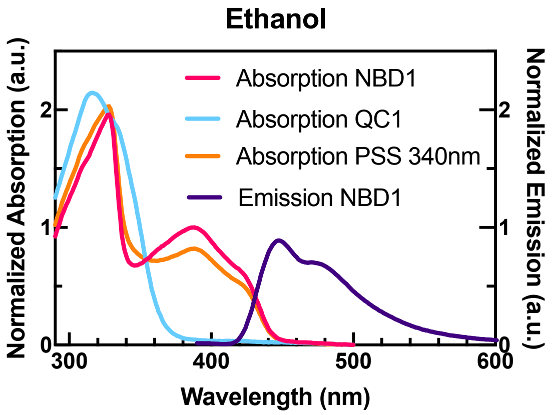

Figure S4: Absorption spectra of NBD1, QC1 (obtained by irradiation at 405 nm), and the photostationary state (irradiation at 340 nm). Emission spectrum of NBD1. All spectra recorded in ethanol.

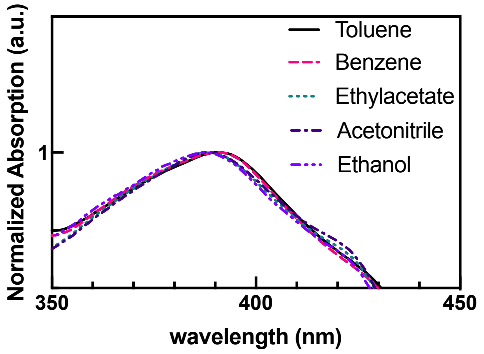

Figure S6: Absorption spectra of NBD1 in different solvents. The spectra are slightly blue shifted in more polar solvents.

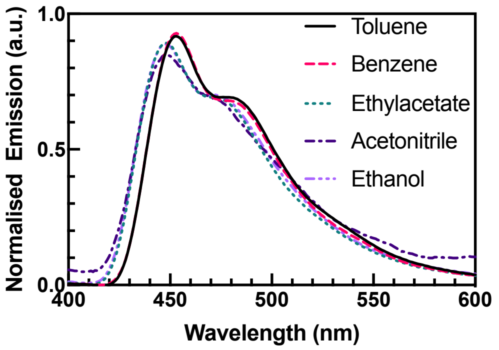

Figure S5 Emission profiles in different solvents. The spectra are blue shifted in more polar solvents.

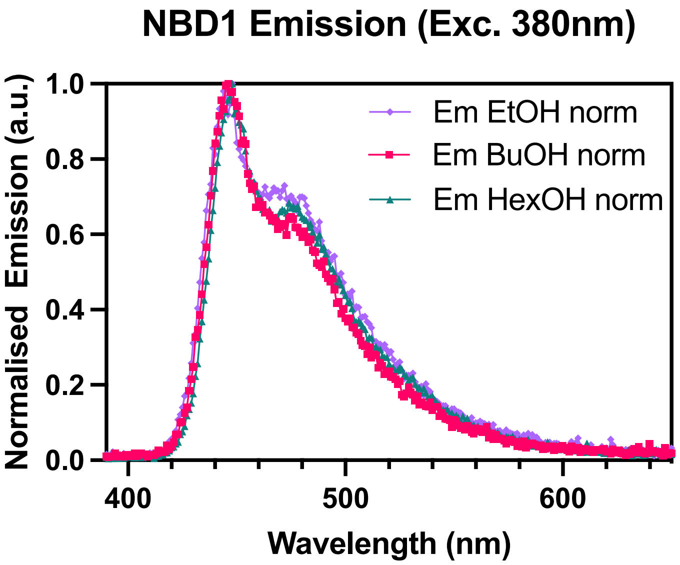

Figure S7: Emission profiles of NBD1 in ethanol, butanol and hexanol.

4. Tissue samples preparations:

Mice tissues were provided by Dr. Dag Sehlin at Uppsala University. Transgenic AD mice carrying the Swedish mutation in APP (tgAPP$_{SWE}$) were reared *ad libitum* at an animal facility at Uppsala University under a 12/12 light cycle. Female, 18- or 21-months old animals were anesthetized with isoflurane and sacrificed by decapitation. The brains were dissected within 3 min post mortem and frozen on dry ice.

Animal procedures were approved by an ethical committee and performed in compliance with national and local animal care and use guidelines (DNr #C17⁄14 at Uppsala University).

Frozen tissue sections of 12 μm thickness were cut in a cryostat microtome (Leica CM 1520, Leica Biosystems, Nussloch, Germany) at -18°C, collected on frosted microscope glasses (VWR) for fluorescence imaging or mounted on conductive ITO glasses (Bruker Daltonics, Bremen, Germany) for MALDI IMS. They were stored at -20°C and they were thawed under vacuum for up to 1 hour prior to performing the experiments.

5. Fluorescent Staining procedures:

- **NBD1**

A NBD1 saturated solution in EtOH was prepared as followed: 0.64 mg were added to 10 mL of EtOH (which would give a solution of 100 μM). The mixture was sonicated for 3 hours, and afterward it was centrifuged at 3000 rpm for 5 min. The obtained saturated solution was decanted to a new vial.
The glasses bearing the sections were immersed in EtOH (1min), EtOH 70% in MQ H_2O (1 min) and MQ H_2O or PBS (5 min), followed by 10 seconds in EtOH 70%. The samples were then positioned in a humidified chamber, 200 μL of saturated solution of NBD1 in EtOH were added on top of the tissue, and let rest for 2 hours in dark. Afterward, the samples were washed in EtOH (10 s) and MQ H_2O (3 x 1 min) and dried in an exicator for at least 30 minutes.

- **Immunohistochemistry**

Brain sections collected on frost glasses were fixed in ice cold 95% ethanol, 70% ethanol and 1xPBS at room temperature. The sections were then blocked with Bovine Serum Albumin (BSA), Normal Goat Serum (NGS) and Triton in 0.1% PBST for 90 minutes at room temperature. The sections were incubated with Aβ antibody 6E10 (mouse, dilution 1:500 in NGS and 0.2% PBST) for over 18 hours at 4°C. Sections were washed with 0.1% PBST and incubated with the secondary antibody (Alexa-flour 488, goat anti mouse, 1:1000) for 60 min at room temperature, and then washed with PBS (3 x 5 min). Sections were finally mounted with DAKO fluorescent mounting media and let rest for 24 hours at room temperature.

6. MALDI MS Imaging:

Before MALDI MSI analysis, the sections mounted on ITO glasses were thawed and dried in desiccator. A series of sequential washed of 100% EtOH (60 s), 70% EtOH (30 s), Carnoy's fluid (6:3:1 EtOH/$CHCl_3$/acetic acid) (90 s), 100% EtOH (15 s), H_2O with 0.2% TFA (60 s), and 100% EtOH (15 s) was carried out. Tissue was subjected to formic acid vapor for 20 min. 2,5-Dihydroxyacetophenone (2,5-DHAP) was applied using an HTX TM-Sprayer (HTX Technologies LLC, Carrboro, NC, USA). A matrix solution of 15 mg/mL 2,5-DHAP in 70% ACN/2% CH_3COOH/2% TFA was sprayed onto the tissue sections using the following instrumental parameters: nitrogen flow (10 pounds per square inch), spray temperature (75 ℃), nozzle height (40 mm), seven passes with offsets and rotations, and spray velocity (1000 mm/min), and isocratic flow of 100 µl/min using 70% CAN as pushing solvent.

MALDI IMS experiments were performed on a rapifleX MALDI time-of-flight (TOF) instrument (Bruker Daltonics). Measurements were performed at 10-µm spatial resolution, with the laser operation at a frequency of 10kHz, a laser power of 90%, and 200 shots per pixel. Data were acquired in linear positive mode in the mass range of 1500 to 6000 *m/z*. Preacquisition calibration was performed using combination of peptide calibration standard I and protein calibration standard I. Acquisition and subsequent processing were performed in flexImaging (version 5.1, Bruker Daltonics).

7. Fluorescence imaging:

Widefield fluorescence microscopy images were acquired with a Zeiss Axiolab 5 equipped with three fluorescence LED channels at 365nm, 470nm, 530nm in combination with filter set 109. 365nm LED was used to visualize NBD1 staining.

Confocal and hyperspectral images were collected on an ELYRA PS.1/LSM 780 laser scanning microscopy equipped with a 32-Channel GaAsP spectral detector (Zeiss) and two PMTs. The objective used was Plan-Apochromat 20×/0.8 (WD = 0.55 mm), ∞/0.17. The excitation wavelength used for NBD1 was 405 nm. The emission spectra were acquired between 415–600 nm.

Photoisomerization experiments were performed using an inverted Leica SP5 MP confocal microscope equipped with a Leica HyD hybrid detector at the Ibima-Plataforma Bionand institute in Malaga. Images were processed with the Zen Black software (Zeiss) and ImageJ.

8. Confocal hyperspectral imaging:

Hyperspectral images were collected with a Zeiss ELYRA PS.1/LSM 780 confocal microscope. A GaAsP multichannel spectral detector allowed collection of image signals at regular intervals of 9 nm between 400 and 600 nm, providing spectral information on each pixel of the image. Irradiation was done at 405 nm, and signal detection every 9 nm between 414 and 600 nm. Spectral unmixing was performed on Zeiss black software, with 3 hand selected 5px areas, or ACE with 3 components.

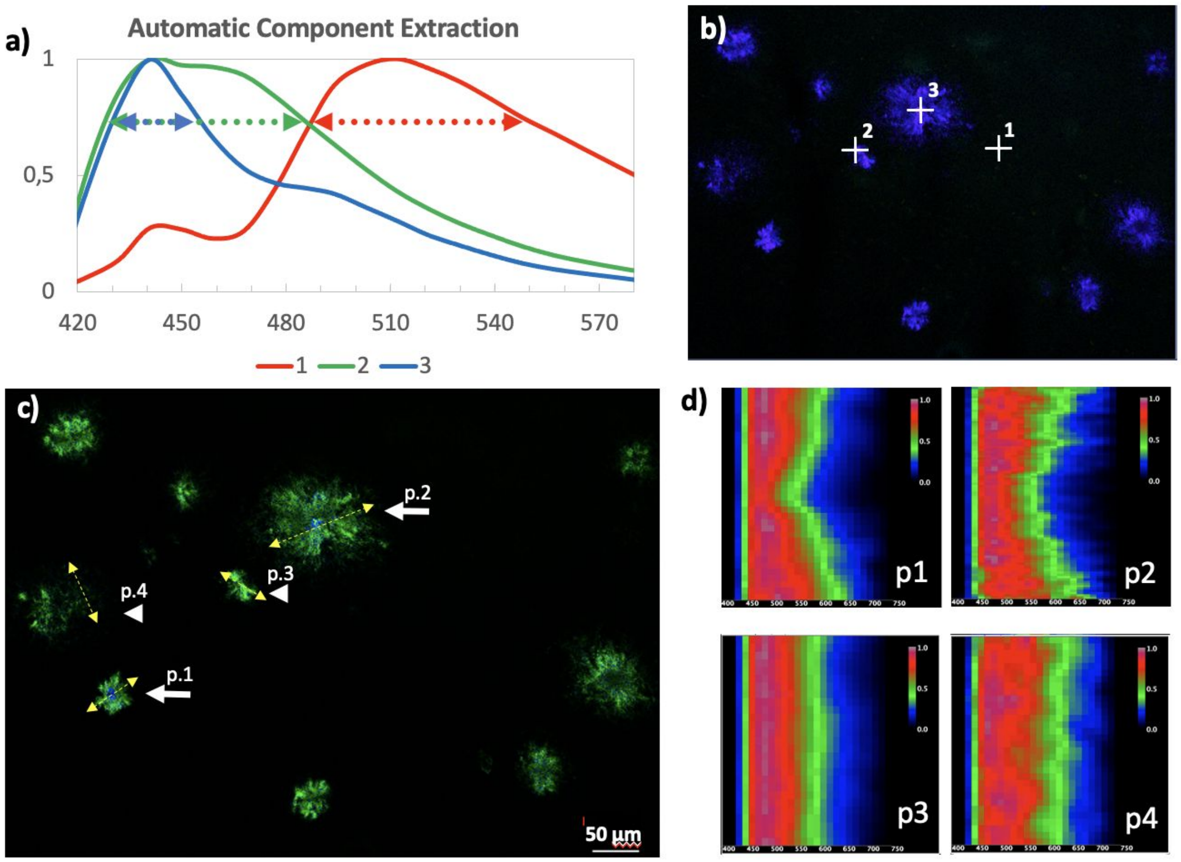

Figure S8: Automatic Component Extraction done with 3 components, their spectra (a) and localization (b). c) Unmixing of the selected area, showing in blue the dense cores of matured cored plaques (p1 and p2). D)Linear scan analyses of p1-4.

9. Photoisomerization and back conversion *in situ*

Photoisomerization experiments were done by means of strong irradiations at 405 every 5 seconds followed by image acquisition over two spectral ranges: 410 – 440 nm and 455 – 465 nm, corresponding to the main emission peaks of NBD1 in tissue. In a first experiment the plaques signal was completely bleached after 20 minutes. The

recovery of the signals was followed first by imaging every 5 minutes for 75 minutes, and then every 60 minutes until reaching 1000 minutes.

To compare signals of different areas Min-Max normalization was performed according to the following equation:

$$x_2 = \frac{(x_1 - x_{min})}{(x_{max} - x_{min})}$$

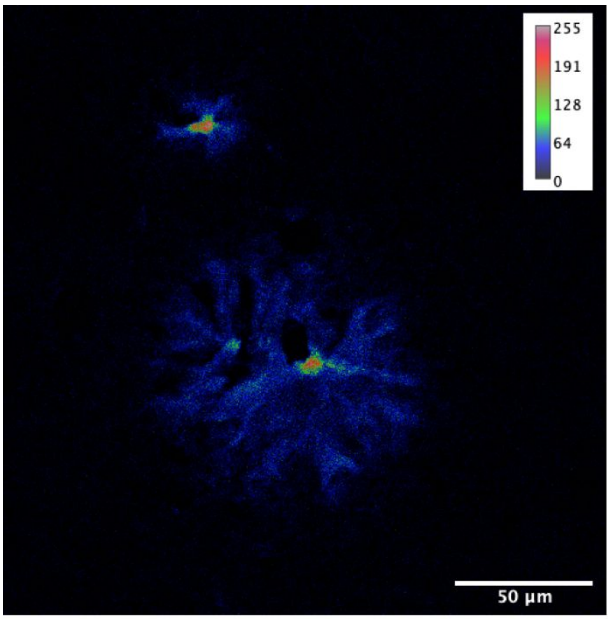

Figure S9 NBD1 stained TgAPPSwe mouse brain section (18m female). Irradiated are in photobleaching experiment 1, containing two cored plaques, LUT "RGB rainbow". Blue areas are identified as plaques periphery, red areas are identified as cores.

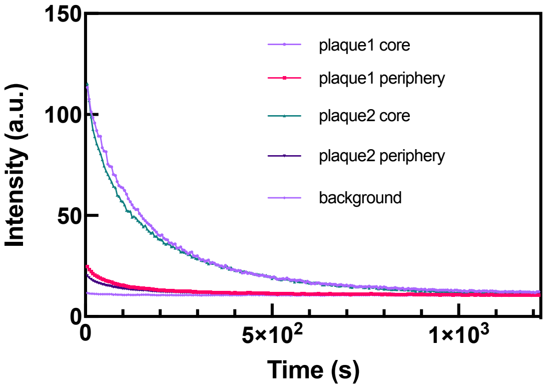

Figure S10 Intensity profiles over time during irradiation at 405 nm of averaged signals in the identified areas of cores and periphery of the plaques.

In a second experiment everything was performed as previously explained, but the photoisomerization was stopped after 160 seconds, when the initial intensity was reduced to about 50%, in order to evaluate degradation effects.

The irradiated area is shown in the following figure S4 and it contains 3 cored plaques, where one is a mature plaque with a very dense core, and the other two are smaller plaques with less intense core signals. This can be seen in the intensity profiles over the plaques, in Figure S5 and S6. Interestingly, the fading rates correlate with the intensity, where core of plaque 1 fades at the slowest rate, cores of plaques 2 and 3 fade at a faster rate, and peripheries fade at the fastest rate, see Figure S7.

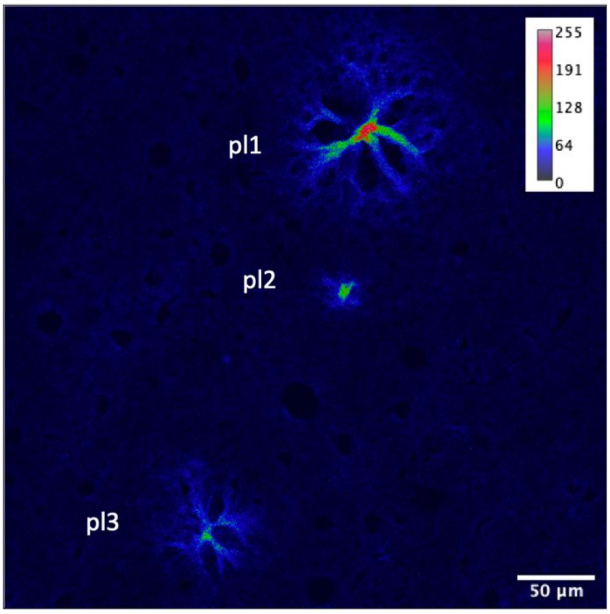

Figure S11 Mouse TgAPPSwe brain tissue (18m, female) visualized using confocal imaging. Irradiated area in photobleaching experiment 2, containing 3 cored plaques, LUT "RGB rainbow". Red area in plaque 1, green areas in plaques 2 and 3 are determined as cores; lighter blue areas are identified as periphery.

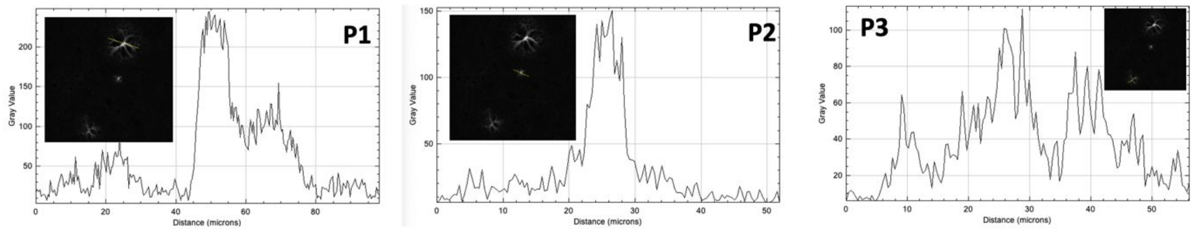

Figure S12 Intensity profiles over space of plaques 1-3. Plaque 1 reach up to 250 in intensity, plaques 2 and 3 max intensities are below 150.

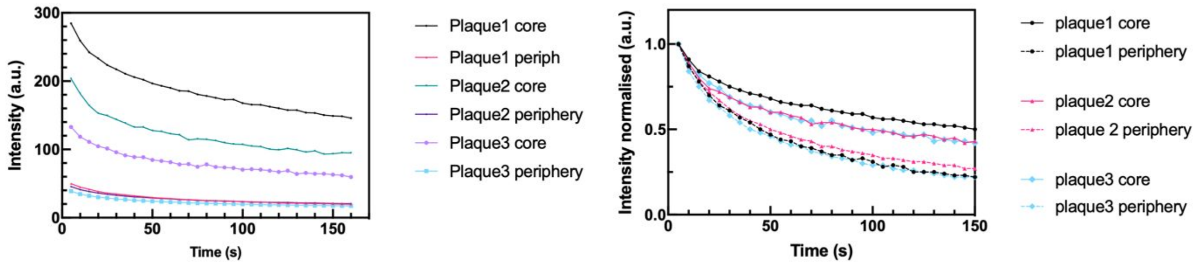

Figure S13: Intensity profiles over time of fluorescence signals of different areas, not normalized (left) and normalized (right)

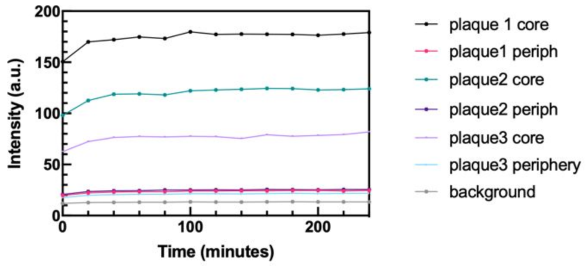

Figure S14 Fluorescence intensity profiles over time averaged over the selected areas showing recovery of the fluorescence signal.

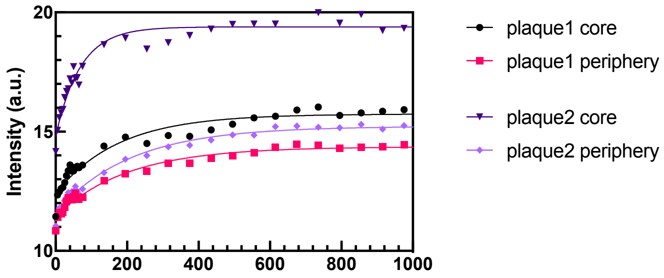

Figure S15 Recovery of fluorescence signal of plaques 1 and 2 (main text, figure 6a) over 1000 minutes.

10. NBD1 and LCO co-staining in TgSWE mice tissue

NBD1 staining was performed as described above. Immediately after tissue washings LCO staining was performed by applying 200 µL of solution containing hFTAA (3µM) and qFTAA (3 µM) to the samples. These were left in a dark humidified chamber for 30 min. Afterward, the samples were washed in MQ H_2O (3 x 5 min.) and dried in an exicator for at least 30 minutes.

The samples were imaged using confocal hyperspectral imaging as described above, and spectral unmixing was performed with Zeiss black software.

The images showed how NBD1 preferably stains highly dense cores of mature plaques, and co-localize with qFTAA stained cores.

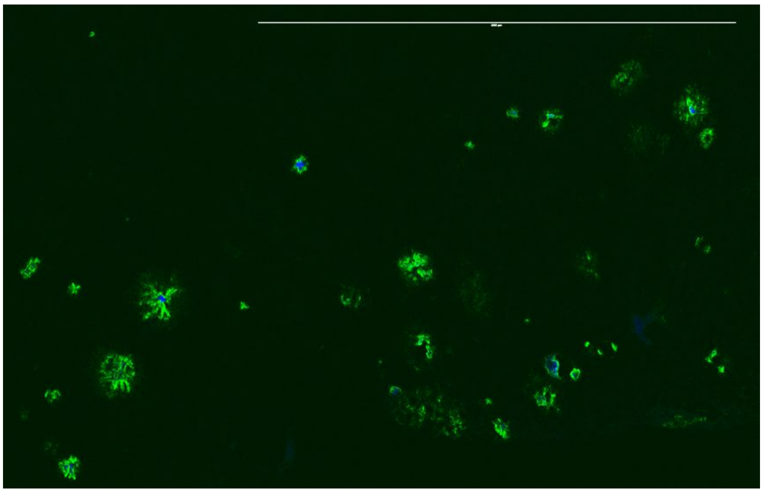

Figure 16 NBD1(blue) and LCO (hFTAA and qFTAA, green) staining in TgSwe mice brain tissue. Confocal imaging, scalebar 1000 um.

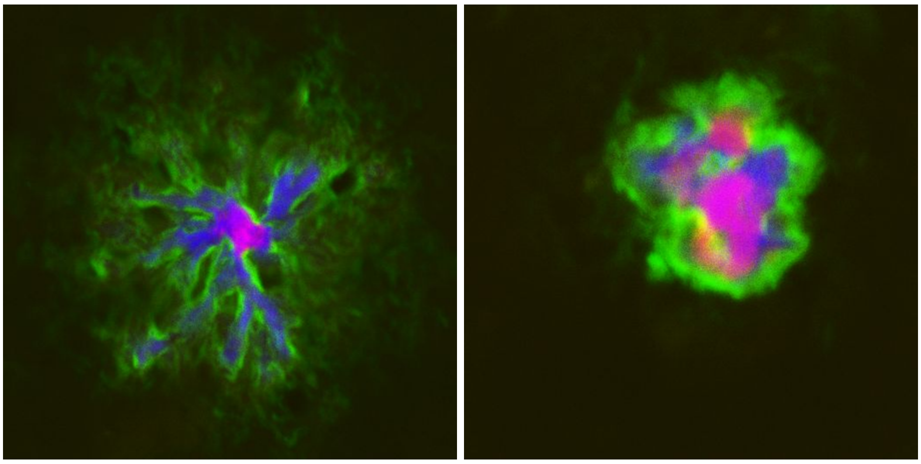

Figure 17 NBD1 and LCO co-staining of amyloid plaques in TgSwe mice brain tissue. Spectral unmixing in of NBD1(purple), hFTAA (green) and qFTAA (blue) showing co-localization of NBD1 staining with qFTAA stained cores.

11. NBD1 staining of sporadic AD human tissue

Microscope glass mounted sections of human tissue with sporadic AD (sAD) were stained with NBD1 with the following procedure:

A NBD1 saturated solution in EtOH was prepared as following: 0.64 mg were added to 10 mL of EtOH (which would give a solution of 100 μM). The mixture was sonicated for 3 hours, and afterward it was centrifuged at 3000 rpm for 5 min. The obtained saturated solution was decanted to a new vial.

The glasses bearing the sections were immersed in EtOH (10 min), EtOH 70% in PBS (10 min) and PBS (10 min), followed by 10 seconds in EtOH 70%. The samples were then positioned in a humidified chamber, 200 μL of saturated solution of NBD1 in EtOH

were added on top of the tissue, and let rest for 3.5 hours in dark. Afterward, the samples were washed in EtOH (10 s) and PBS (3 x 5 min) and dried in an exicator for at least 30 minutes.

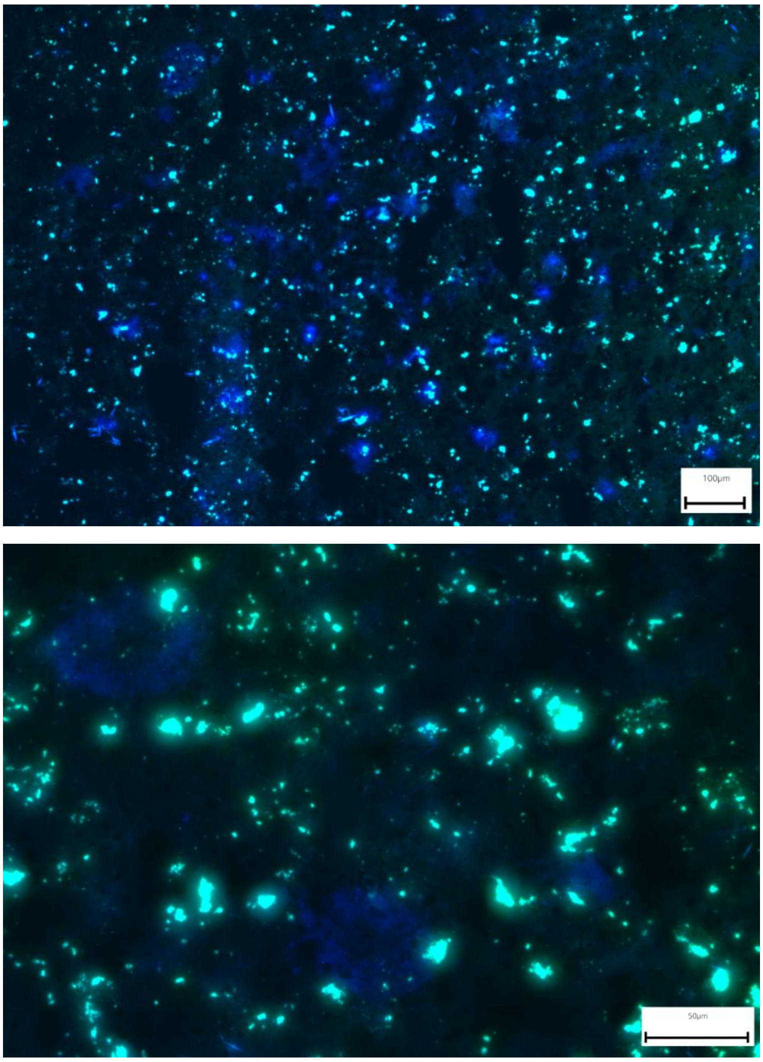

Figure S18 Widefield imaging of NBD1 stained sAD human tissue. Amyloid plaques are visible in the blue channel.

12. Molecular Docking

The molecular docking was performed using the open-source program AutoDock Vina v1.1.2.[1] The structures of amyloid aggregates were obtained from RCSB Protein Data Bank (PDB ID: 2LMQ[2] for the Aβ40 and PDB ID: 5OQV[3] for the Aβ42). The docking simulations were carried out with a box centered on the Aβ oligomers and an adequate grid to contain the entire receptor. The number of cpus employed were 4, with an exhaustiveness of 1000 and a maximum number of binding modes to generate of 100. The other parameters of AutoDock Vina were left at default settings. The interpretation

of the results was done with the AutoDock Tools 4.2 software[4] and the obtained complexes were ranked considering their docking scores. 2D diagrams of the interactions between the ligand and the receptor were done with the software Discovery Studio Visualizer v.21.1.0.20298. (BIOVIA, Dassault Systèmes, Discovery Studio Visualizer, v.21.1.0.20298, San Diego: Dassault Systèmes, 2021).

Table S1: Analysis of the close contacts between NBD1 and the amyloid fibrils: Aβ1-40 (PDB ID: 2LMQ) and Aβ1-42 (PDB ID: 5OQV) (legend: A:G37 is the Glycine 37 in the chain A).

Amino acids	Gly<br>G	Ala<br>A	Val<br>V	Leu<br>L	Ile<br>I	Phe<br>F	Asn<br>N	Lys<br>K
**$A\beta_{1-40}$**								
**2LMQ**<br>($E_{binding}$ = -11.9 kcal/mol)	A:G37	A:A21<br>A:A30<br>B:A21<br>D:A21	A:V36<br>C:V24	A:L34<br>B:L17<br>C:L17	A:I32<br>B:I32<br>C:I32<br>D:I32	B:F19<br>C:F19<br>D:F19		A:K28
**$A\beta_{1-42}$**								
**5OQV**<br>($E_{binding}$ = -10.0 kcal/mol)	A:G29<br>C:G29<br>E:G29		E:V18		A:I31	A:F19<br>C:F19<br>E:F19<br>E:F20	C:N27<br>E:N27	E:K16<br>E:K28

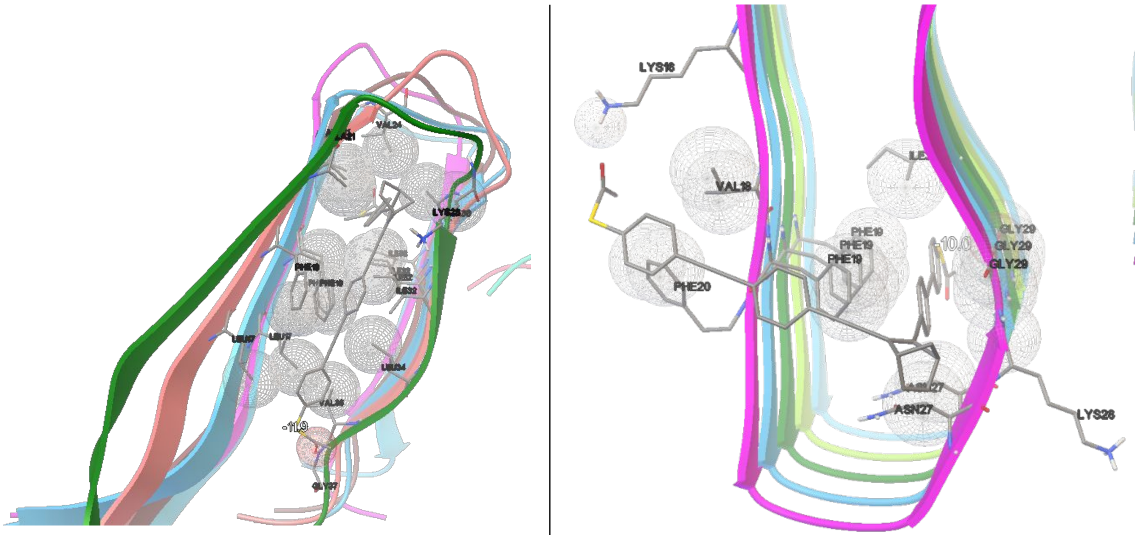

2LMQ+NBD1 **5OQV+NBD1**

Figure S19: Close contacts between NBD1 interacting with the amyloid fibrils 2LMQ and 5OQV. The receptors structures are coloured by chains and for the ligand (NBD1) the carbons are in grey, oxygens in red and sulphurs in yellow.

References:

1 Trott, O. and Olson, A.J. AutoDock Vina: Improving the speed and accuracy of docking with a new scoring function, efficient optimization, and multithreading. J. Comput. Chem., **2010**, 31: 455-461. https://doi.org/10.1002/jcc.21334

2 Paravastu, A. K.; Leapman, R. D.; Yau, W. M.; Tycko, R. Molecular Structural Basis for Polymorphism in Alzheimer's β-Amyloid Fibrils. Proc. Natl. Acad. Sci. U. S. A. **2008**, 105 (47), 18349–18354. https://doi.org/10.1073/pnas.0806270105.

3 Gremer, L.; Schenk, C.; Reinartz, E.; Ravelli, R. B. G.; Tusche, M.; Lopez-iglesias, C.; Hoyer, W.; Heise, H.; Willbold, D. Fibril Structure of Amyloid-β(1–42) by Cryo–Electron Microscopy. Science **2017**, 119, 116–119.

4 Morris, G.M., Huey, R., Lindstrom, W., Sanner, M.F., Belew, R.K., Goodsell, D.S. and Olson, A.J., AutoDock4 and AutoDockTools4: Automated docking with selective receptor flexibility. J. Comput. Chem., **2009**, 30: 2785-2791. https://doi.org/10.1002/jcc.21256
